# Placental Abnormalities and Placenta-Related Complications Following *In-Vitro* Fertilization: Based on National Hospitalized Data in China

**DOI:** 10.3389/fendo.2022.924070

**Published:** 2022-06-30

**Authors:** Fei Kong, Yu Fu, Huifeng Shi, Rong Li, Yangyu Zhao, Yuanyuan Wang, Jie Qiao

**Affiliations:** ^1^ Center for Reproductive Medicine, Department of Obstetrics and Gynecology, Peking University Third Hospital, Beijing, China; ^2^ National Clinical Research Center for Obstetrical and Gynecology, Peking University Third Hospital, Beijing, China; ^3^ Key Laboratory of Assisted Reproduction (Peking University), Ministry of Education, Beijing, China; ^4^ National Center for Healthcare Quality Management in Obstetrics, Peking University Third Hospital, Beijing, China

**Keywords:** *in-vitro* fertilization, placental abnormality, placenta-related complication, maternal complication, neonatal complication

## Abstract

**Introduction:**

Emerging evidence has shown that *in-vitro* fertilization (IVF) is associated with higher risks of certain placental abnormalities or complications, such as placental abruption, preeclampsia, and preterm birth. However, there is a lack of large population-based analysis focusing on placental abnormalities or complications following IVF treatment. This study aimed to estimate the absolute risk of placental abnormalities or complications during IVF-conceived pregnancy.

**Methods:**

We conducted a retrospective cohort study of 16 535 852 singleton pregnancies with delivery outcomes in China between 2013 and 2018, based on the Hospital Quality Monitoring System databases. Main outcomes included placental abnormalities (placenta previa, placental abruption, placenta accrete, and abnormal morphology of placenta) and placenta-related complications (gestational hypertension, preeclampsia, eclampsia, preterm birth, fetal distress, and fetal growth restriction (FGR)). Poisson regression modeling with restricted cubic splines of exact maternal age was used to estimate the absolute risk in both the IVF and non-IVF groups.

**Results:**

The IVF group (n = 183 059) was more likely than the non-IVF group (n = 16 352 793) to present placenta previa (aRR: 1.87 [1.83–1.91]), placental abruption (aRR: 1.16 [1.11–1.21]), placenta accrete (aRR: 2.00 [1.96–2.04]), abnormal morphology of placenta (aRR: 2.12 [2.07 to 2.16]), gestational hypertension (aRR: 1.55 [1.51–1.59]), preeclampsia (aRR: 1.54 [1.51–1.57]), preterm birth (aRR: 1.48 [1.46–1.51]), fetal distress (aRR: 1.39 [1.37–1.42]), and FGR (aRR: 1.36 [1.30–1.42]), but no significant difference in eclampsia (aRR: 0.91 [0.80–1.04]) was found. The absolute risk of each outcome with increasing maternal age in both the IVF and non-IVF group presented two patterns: an upward curve showing in placenta previa, placenta accreta, abnormal morphology of placenta, and gestational hypertension; and a J-shape curve showing in placental abruption, preeclampsia, eclampsia, preterm birth, fetal distress, and FGR.

**Conclusion:**

IVF is an independent risk factor for placental abnormalities and placental-related complications, and the risk is associated with maternal age. Further research is needed to evaluate the long-term placenta-related chronic diseases of IVF patients and their offspring.

## Introduction

The placenta is the first organ to form during mammalian embryogenesis. As the vascular interface between maternal and fetal circulation systems, the placenta plays a crucial role in nutrient, gas, immune, and toxic substances transfers or barriers between mothers and fetuses (1). Hence, placental development and function are essential for developing mammalian embryos in the uterine environment. However, if the placental development or function is impaired, or the capacity to adapt to adverse environmental exposures is exceeded, intrauterine fetal development and long-term health in childhood and even adulthood may be compromised. Such short- and/or long-term adverse outcomes include: i. placental abnormalities, such as placenta previa, placental abruption, placenta accreta, and abnormal placental morphogenesis; ii. placenta-related complications during prenatal and perinatal periods, such as preeclampsia, fetal growth restriction, preterm birth, fetal distress, and fetal growth restriction (FGR) (2, 3); and iii. placenta-related chronic diseases in adulthood, such as cardiovascular disease, diabetes, and obesity (4). Therefore, the placenta has been considered as a key predictor of maternal and offspring’s health.

Over the past four decades, there has been a growing appreciation that *in-vitro* fertilization (IVF) has helped millions of infertile couples worldwide to have babies. The latest estimate of global utilization is 2.8 million IVF cycles and 0.9 million IVF babies in 2012 (5). In 2016, China reported 0.9 million IVF cycles and 0.3 million IVF babies, which accounted for 1.6% of the total live births (6). The embryo implantation and development, which is mainly dependent on the placental formation and function, is critical to the success of IVF. Some previous studies reported that IVF-conceived pregnancies had thicker and heavier placentas, higher placental weight/birthweight ratio, decreased apical microvilli, and increased multiple vacuoles compared with spontaneous pregnancies, suggesting maternofetal traffic downregulation following IVF treatment (7, 8). Blastocyst malrotation at implantation in IVF treatment may lead to low-lying placenta, placenta previa, and velamentous insertion of the umbilical cord (9). Furthermore, IVF has been associated with higher risks of certain placental abnormalities or complications, such as placental abruption, preeclampsia, and preterm birth (10–12). However, there is a lack of population-based analysis focusing on placental abnormalities or complications after IVF treatment.

Based on a retrospective analysis of 17 million hospitalized deliveries between 2013 and 2018 in China, this study aimed to estimate the absolute risk of placental abnormalities or complications following IVF treatment to provide evidence for medical professionals to make optimal decisions in clinical management, thereby improving maternal and offspring’s safety and health.

## Methods

### Study Design and Population

**Figure 1:**
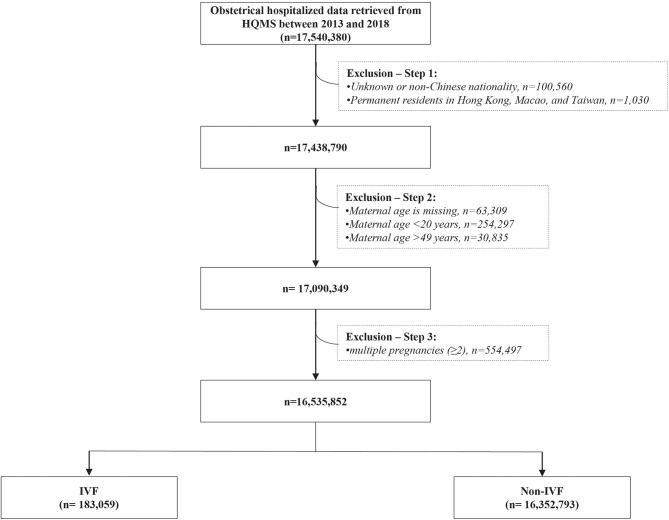
The flowchart of eligible study population in this study IVF, *in vitro* fertilization; Non-IVF, non-*in vitro* fertilization.

This was a retrospective cohort study of 17 million hospitalized delivery cases in 1853 maternity hospitals between 2013 and 2018 in all 31 provinces of Mainland China. 17 540 380 hospitalized delivery cases were retrieved from the Hospital Quality Monitoring System (HQMS) databases, established and formally run by National Health Commission of China since 2013. After excluding 1 004 528 (5.72%) cases with unknown or non-Chinese nationality (100 560 cases), permanent residents in Hong Kong, Macao, and Taiwan (1 030 cases), missing value of maternal age (63 309 cases), maternal age below 20 years (254 297 cases) or beyond 49 years (30 835), and multiple pregnancies (554 497 cases), there were 16 535 852 eligible cases included in the analysis, including 183 059 IVF-conceived pregnancies (the IVF group) and 16 352 793 spontaneous pregnancies (the non-IVF group) (**Figure 1**).

### Variables

In the HQMS database, the standardized electronic hospitalized discharge records (also known as “first page of electronic medical record”) were automatically collected from each hospital. All diagnoses and operating procedures were coded by using the International Classification of Diseases -10th Revision (ICD-10) and the International Classification of Diseases -9th Revision, Clinical Modification (ICD-9-CM) (**Table S1**) (13, 14). In this study, we mainly used three categories of variables for each pregnant woman (1): general characteristics: geographical region (eastern, central, or western), year of discharge, maternal age at delivery, and ethnicity (Han or Minority) (2); chronic diseases before pregnancy: chronic hypertension, diabetes, thyroid diseases, anemia, circulatory diseases and other chronic conditions (including coagulation disorders, kidney diseases, diseases of connective tissues, respiratory diseases, and digestive system diseases) (3); placental abnormalities: placenta previa, placental abruption, placenta accrete, and abnormal morphology of placenta (4); placenta-related complications: gestational hypertension, preeclampsia, eclampsia, preterm birth, fetal distress, and fetal growth restriction (FGR).

### Statistical Analysis

Chi-squared test for categorical variables and Student’s t-test for continuous variables were performed to compare the differences of general characteristics between the IVF group and the non-IVF group. Considering maternal age usually presents an independent, nonlinear association with many maternal and neonatal complications (15), we performed Poisson regression models with restricted cubic splines of exact maternal age to allow the most flexible characterization of the nonlinear association between maternal age and each placenta-related outcome. For each restricted cubic spline of maternal age, the number of knots placed at the default percentiles was identified by the principle of minimized Akaike Information Criterion (AIC) (16, 17). For each placenta-related outcome, we performed two Poisson regression models: an unadjusted model with only maternal age spline terms to examine crude relative risk (RR) and 95% confidence interval (CI) of IVF vs. non-IVF (2); a multivariable model adjusted by general characteristics and chronic diseases before pregnancy to examine adjusted RR (aRR_1_) and 95% CI of IVF vs. non-IVF. Finally, we used the multivariable model to calculate the predicted absolute risk (probability and 95% CI). We then graphically presented them to visually illustrate the absolute risk of each placenta-related complication by maternal age. All analyses were conducted using the SAS 9.0 software (18). A two-tailed P value < 0.05 was considered statistically significant. Sensitivity analyses of this study were done by comparing the results of two Poisson regression models (unadjusted model and multivariable model) with restricted cubic splines of exact maternal age to assess for confounding from general characteristics, chronic diseases before pregnancy, and year-by-year maternal age. We also performed an additional sensitivity analysis by using logistic regression models with restricted cubic splines of exact maternal age, with adjustment for geographic region, maternal age, year, ethnicity, and chronic diseases before pregnancy.

## Results

In this study, there were 183 059 pregnancies in the IVF group (1.1%) and 16 352 793 pregnancies in the non-IVF group (98.9%). The differences in general characteristics between the IVF and non-IVF groups were statistically significant (**Table 1**). The proportion of pregnant women with advanced maternal age (≥35 years) was markedly higher in the IVF group (31.9%) than that of the non-IVF group (13.7%). Compared with the non-IVF group, the IVF group was more likely to have chronic diseases before pregnancy, including chronic hypertension, diabetes, thyroid diseases, anemia, circulatory diseases, and other chronic conditions. Thus, these general characteristics of the study population would be adjusted in the multivariable modeling in order to control confounding bias.

**Table 1:** The general characteristics of study population in this study.

General characteristics	Total (n = 16,535,852)	IVF (n = 183,059)	non-IVF (n = 16,352,793)	*P*-value
**Geographical area**				
Eastern	8462641 (51.2)	101253 (55.3)	8361388 (51.1)	<0.001
Central	3852875 (23.3)	43965 (24.0)	3808910 (23.3)	<0.001
Western	4220336 (25.5)	37841 (20.7)	4182495 (25.6)	<0.001
**Year**				
2013	751997 (4.5)	5477 (3.0)	746520 (4.6)	<0.001
2014	1438167 (8.7)	9791 (5.4)	1428376 (8.7)	<0.001
2015	1327773 (8)	13383 (7.3)	1314390 (8.0)	<0.001
2016	4370506 (26.4)	41992 (22.9)	4328514 (26.5)	<0.001
2017	4451031 (26.9)	47874 (26.2)	4403157 (26.9)	<0.001
2018	4196378 (25.4)	64542 (35.3)	4131836 (25.3)	<0.001
**Maternal age at birth, Mean (SD)**	29.20 (4.6)	32.51 (4.5)	29.16 (4.6)	<0.001
20-24	2419814 (14.6)	4859 (2.7)	2414955 (14.8)	<0.001
25-29	7184983 (43.5)	43033 (23.5)	7141950 (43.7)	<0.001
30-34	4641920 (28.1)	76727 (41.9)	4565193 (27.9)	<0.001
35-39	1857960 (11.2)	46330 (25.3)	1811630 (11.1)	<0.001
40-44	402996 (2.4)	10990 (6.0)	392006 (2.4)	<0.001
45-49	28179 (0.2)	1120 (0.6)	27059 (0.2)	<0.001
**Ethnicity**				
Minority	1515065 (9.2)	16366 (8.9)	1498699 (9.2)	<0.001
Han	14856494 (89.8)	165736 (90.5)	14690758 (89.8)	<0.001
Unknown	164293 (1.0)	957 (0.5)	163336 (1.0)	<0.001
**Chronic diseases before pregnancy**				
Chronic hypertension	133051 (0.8)	2713 (1.5)	130338 (0.8)	<0.001
Diabetes	158200 (1.0)	3822 (2.1)	154378 (0.9)	<0.001
Thyroid diseases	640093 (3.9)	13441 (7.3)	626652 (3.8)	<0.001
Anemia	2589684 (15.7)	29171 (15.9)	2560513 (15.7)	0.0012
Circulatory diseases	167004 (1.0)	2665 (1.5)	164339 (1.0)	<0.001
*Other chronic conditions	734827 (4.4)	11226 (6.1)	723601 (4.4)	<0.001

^*^ “Other chronic conditions” included coagulation disorders, kidney diseases, diseases of connective tissues, diseases of respiratory system, and diseases of digestive system.

After adjusting for general characteristics and chronic diseases before pregnancy with restricted cubic splines of exact maternal age (**Table 2**), the IVF group was more likely than the non-IVF group to present each outcome of placental abnormalities: placenta previa (4.8% vs 2.0%; aRR=1.87, 95% CI: 1.83 to 1.91), placental abruption (1.0% vs 0.8%; aRR=1.16, 95% CI: 1.11 to 1.21), placenta accrete (4.7% vs 2.0%; aRR=2.00, 95% CI: 1.96 to 2.04), abnormal morphology of placenta (4.5% vs 1.8%; aRR=2.12, 95% CI: 2.07 to 2.16) (**Table S2**).

**Table 2:** Associations of IVF with placental abnormalities and placenta-related complications.

	Total (n = 16,535,852)	IVF (n = 183,059)	non-IVF (n = 16,352,793)	RR (95% CI)	aRR (95% CI)
**Placental abnormalities**					
Placenta previa	328773 (1.99)	8865 (4.8)	319908 (2.0)	**2.48 (2.42 to 2.53)**	**1.87 (1.83 to 1.91)**
Placental abruption	127427 (0.77)	1853 (1.0)	125574 (0.8)	**1.32 (1.26 to 1.38)**	**1.16 (1.11 to 1.21)**
Placenta accreta	326875 (1.98)	8554 (4.7)	318321 (2.0)	**2.40 (2.35 to 2.45)**	**2.00 (1.96 to 2.04)**
Abnormal morphology of placenta	308626 (1.87)	8231 (4.5)	300395 (1.8)	**2.45 (2.40 to 2.50)**	**2.12 (2.07 to 2.16)**
**Placenta-related complications**					
Gestational hypertension	276136 (1.67)	5962 (3.3)	270174 (1.7)	**1.97 (1.92 to 2.02)**	**1.55 (1.51 to 1.59)**
Preeclampsia	456977 (2.76)	10082 (5.5)	446895 (2.7)	**2.02 (1.98 to 2.05)**	**1.54 (1.51 to 1.57)**
Eclampsia	20621 (0.12)	226 (0.1)	20395 (0.1)	0.99 (0.87 to 1.13)	0.91 (0.80 to 1.04)
Preterm birth	808063 (4.89)	15066 (8.2)	792997 (4.9)	**1.70 (1.67 to 1.72)**	**1.48 (1.46 to 1.51)**
Fetal distress	1010414 (6.11)	14627 (8.0)	995787 (6.1)	**1.31 (1.29 to 1.33)**	**1.39 (1.37 to 1.42)**
FGR	134722 (0.81)	2205 (1.2)	132517 (0.8)	**1.49 (1.43 to 1.55)**	**1.36 (1.30 to 1.42)**

IVF, in vitro fertilization; Non-IVF, non-in vitro fertilization; FGR, fetal growth restriction. RR, relative risk; aRR, adjusted relative risk. CI, confidence interval. RR were calculated in Poisson regression modelling with restricted cubic splines of exact maternal age. aRR were calculated in Poisson regression modelling with restricted cubic splines of exact maternal age, with adjustment for geographic region, maternal age, year, ethnicity, and chronic diseases before pregnancy.

The bold values means has statistically significant.

Except for eclampsia, the IVF group was more likely to present all the other placenta-related complications than the non-IVF group: gestational hypertension (3.3% vs 1.7%; aRR_1 =_ 1.55, 95% CI: 1.51 to 1.59), preeclampsia (5.5% vs 2.7%; aRR_1 =_ 1.54, 95% CI: 1.51 to 1.57), preterm birth (8.2% vs 4.9%; aRR_1 =_ 1.48, 95% CI: 1.46 to 1.51), fetal distress (8.0% vs 6.1%; aRR_1 =_ 1.39, 95% CI: 1.37 to 1.42), and FGR (1.2% vs 0.8%; aRR_1 =_ 1.36, 95% CI: 1.30 to 1.42) (**Table 2**). After further adjusting for the four placental abnormalities above, the IVF group continued to present a higher risk of these placenta-related complications comparing to the non-IVF group: gestational hypertension (aRR_2 =_ 1.54, 95% CI: 1.50 to 1.58), preeclampsia (aRR_2 =_ 1.52, 95% CI: 1.49 to 1.55), preterm birth (aRR_2 =_ 1.34, 95% CI: 1.32 to 1.36), fetal distress (aRR_2 =_ 1.38, 95% CI: 1.36 to 1.40), and FGR (aRR_2 =_ 1.26, 95% CI: 1.21 to 1.32) (**Table 2**).

**Figure 2:**
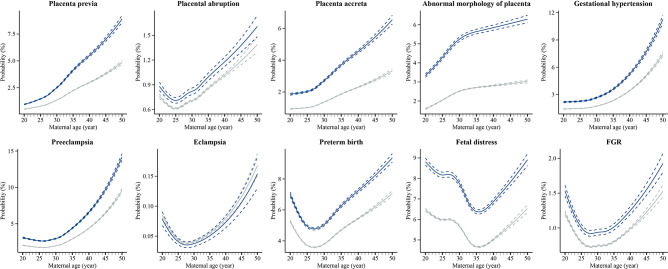
Estimated absolute risks of placental abnormalities and placenta-related complications at each maternal age in IVF group and non-IVF group IVF, *in vitro* fertilization; Non-IVF, non-*in vitro* fertilization; FGR, fetal growth restriction. Estimated absolute risks (probabilities) were calculated by using Poisson regression modelling with restricted cubic splines of exact maternal age, with adjustment for geographic region, maternal age, year, ethnicity, and chronic diseases before pregnancy.

**Figure 2** shows the absolute risk and 95% CI of each outcome by maternal age in both the IVF and non-IVF groups. It can be shown that the IVF group had a higher absolute risk of each outcome (except for eclampsia) at each maternal age than the non-IVF group, and the gaps between the two groups demonstrated a growing trend with the increase of maternal age. Furthermore, the curves can be mainly concluded in two patterns. The first pattern was an upward curve, with absolute risk continuing to increase with maternal age, including placenta previa, placenta accreta, abnormal morphology of placenta, and gestational hypertension. The second pattern was a J-shape curve that the absolute risk dropped at first and then increased with maternal age, including placental abruption (the inflection point is 25 years), preeclampsia (26 years), eclampsia (28 years), preterm birth (27 years), fetal distress (35 years), and FGR (28 years). The sensitivity analysis showed that different statistical approaches did not substantially affect the estimates (**Table S3**).

## Discussion

On the basis of national hospitalized data in China, we found that IVF-conceived pregnancies had a higher risk of placenta-related adverse outcomes than non-IVF pregnancies, including four categories of placental abnormalities (placenta previa, placental abruption, placenta accrete, and abnormal morphology of placenta) and five categories of placenta-related complications (gestational hypertension, preeclampsia, preterm birth, fetal distress, and FGR), but no significant association with eclampsia was found. In both the IVF and non-IVF groups, the absolute risk of each outcome was associated with increasing maternal age, presenting two patterns: an upward curve showing in placenta previa, placenta accreta, abnormal morphology of placenta, and gestational hypertension; and a J-shape curve showing in placental abruption, preeclampsia, eclampsia, preterm birth, fetal distress, and FGR.

The findings of this study demonstrated that IVF is an independent risk factor for placenta previa, placental abruption, placental accreta, and abnormal morphology of placenta after adjusting for factors such as maternal age at birth, geographical area, ethnicity, the year of IVF implementation, and chronic diseases before pregnancy, which is consistent with the previous studies. In earlier epidemiologic studies and systematic reviews, IVF is a risk factor of placenta previa (19–22) and placental abruption (23, 24). IVF singleton pregnancies are associated with a higher incidence of placenta accreta and velamentous placenta (25–28). Moreover, we also noted a greater risk for developing gestational hypertension, preeclampsia, preterm birth, fetal distress, and FGR among the IVF group, which has been documented in other studies (29–31). While there is a scientific consensus about the link between IVF and placental abnormalities or complications, current evidence remains fragmented. Our study generated integrated evidence from a large population-based analysis to confirm the hypothesis that IVF is associated with placenta-related adverse outcomes.

We further analyzed the relationship between each placenta-related adverse outcome and maternal age. We detected that the IVF group had a higher absolute risk at each maternal age than the non-IVF group. The absolute risk of each outcome with increasing maternal age in both the IVF and non-IVF groups presented in two dominant patterns. The first was an upward curve indicating that the absolute risk increases with maternal age. The second was a J-shape curve indicating that the absolute risk is higher at the younger age interval (20-24 years or below) and advanced age interval (35 years or above). These findings illustrated that the optimal age for a woman to be pregnant, whether conceived spontaneously or by IVF treatment, is 25-34 years, which has been well established in many pieces of literature (32–35). Meanwhile, many studies have attempted to analyze the biological plausibility of maternal age-related higher absolute risk of adverse obstetrical and perinatal outcomes, including placental abnormalities or complications. Firstly, it is clear that advanced maternal age is an independent strong risk factor, since the majority of adverse outcomes can be explained by the physio-pathological changes in the female reproductive apparatus that come with aging and its inherent comorbidities (36). Placenta senescence is characterized by reduced telomerase activity and aging suppressor expression (37), increased DNA damage and DNA oxidation{Biron-Shental, 2010 #140}{Biron-Shental, 2010 #140}, and increased expression of senescent biomarkers. In recent years, with the rapid increase of the number of older pregnancies, more studies have focused on the relationship between critical placental aging regulation related proteins and placental adverse outcomes. One study showed that advanced maternal age pregnancy can result in reduced α-Klotho (a well-known antiaging protein) expression in placental trophoblasts, which ultimately lead to placental malformation and adverse perinatal outcomes, possibly through the reduction in the transcription of cell adhesion molecule genes (38). In addition, age-related risks of some adverse pregnancy and birth outcomes for women in younger age groups cannot be overlooked. Although more evidence from the perspective of physiological and pathological mechanism is needed to explain and distinguish why some adverse consequences are positively related to young pregnancy while others are negatively related, absolute risk at the younger age interval can be attributed to unwanted pregnancy, inadequate prenatal care, low income and more social factors to some extent (15). In conclusion, our findings provided compelling evidence for both the reproductive and obstetrical practitioners to focus on the prevention of placenta-related adverse outcomes, particularly for women with younger or advanced maternal age who are undergoing IVF.

As for the location and morphology of the placenta, previous investigations have reported that ovulation induction was associated with an increased risk of placental abruption (39). In addition, *in vitro*-fertilized embryos are transferred to the uterine cavity by the transcervical route using a catheter. This procedure may induce uterine contraction, possibly due to the release of prostaglandins after mechanical stimulation of the internal cervical, which could modify the physiological interaction between the embryo and the endometrium and/or intrauterine blood flow, thereby affecting the embryo implantation process (9). IVF covers a variety of complex operations, including ovulation induction, egg retrieval, fertilization method (conventional IVF or intracytoplasmic sperm injection), type of embryo transfer (fresh embryo or frozen embryo, cleavage-stage embryos or blastocysts), and other operations such as maturation of oocytes *in vitro* and preimplantation genetic test (40). These IVF-related operations may have independent or additive effects on the occurrence of placental structure and function abnormalities compared with spontaneous pregnancy, illuminating the importance of examining the complex relationships between IVF-related operations and the described adverse perinatal outcomes.

While our study was not designed to explain the mechanisms through which IVF may adversely impact placental development, emerging studies are searching for the answers since placental abnormalities are associated with pregnancy-related complications, such as preeclampsia, preterm delivery, and intrauterine growth restriction (2). Evidence suggested that while IVF treatment does not have an adverse effect on the chromosomal constitution of fetal and placental lineages (41), 3405 differentially regulated genes were found to be significantly dysregulated from four human placental villi from first-trimester samples obtained from patients undergoing IVF treatment due to oviductal factors (42). The genes were involved in more than 50 biological processes and pathways, including coagulation cascades, immune response, transmembrane signaling, metabolism, cell cycle, stress control, invasion, and vascularization, whose disruption can cause detrimental outcomes (42). One study showed that IVF treatment could alter the placental phospholipid profiles, affecting the flexibility, fluidity, and function of transporting proteins in the membrane (43). Such alternations can then lead to placental complications. In addition to the genetic perturbations, epigenetic alterations, such as changes in DNA methylation patterns, were detected in the placenta and extraembryonic tissues in IVF-conceived mice, leading to nutrient transport and amino acid metabolism dysregulation (44). This could result in compensatory placenta enlargement to meet fetal nutrient requirements at the end of gestation, which in turn may increase the risk of abnormal placental location (45). As the precise underlying mechanisms that explain the impact of IVF on placental development and function remain to be elucidated, the need for continued exploration to accrue insights on placental complications after IVF treatment has become clearer.

China is currently the country with the largest capacity for ART services globally (6). In light of the relaxing population policy, more potential infertile couples in China might have birth plans and thus pose more disease burden after IVF pregnancy. Both the public health and clinical researchers will put more emphasis on the considerable impact of IVF treatment on the short- or long-term health outcomes of pregnant women, fetuses, and offspring. Our study generates preliminary evidence about the risk of placental abnormalities and placenta-related complications following IVF treatment, prompting IVF specialists to heed the disease susceptibility associated with IVF operations and procedures to mitigate relevant disease risk. The current study’s findings also underline the importance of gestation period management strategies of obstetric complications for obstetric clinicians, with special attention to women with younger or advanced maternal age undergoing IVF, from the perspective of maternal and child health.

## Limitations of the Study

This study has some limitations that should be cautiously considered when interpreting the results. First of all, while we attempted to control for several factors, the HQMS database lacks several key obstetric risk factors, such as maternal health-related information before and during pregnancy (e.g., smoking, drinking, or medication use), paternal characteristics, and family history, which may also be confounding factors of the outcome variables. In addition, IVF treatment involves a variety of medications or surgical procedures. Thus, it is necessary to further analyze the impact of specific operations or medication use on the risk of placenta-related outcomes. Last, longitudinal cohort studies are warranted to trace the incidence of placenta-related chronic diseases and examine the long-term health outcomes of IVF patients and their offspring.

## Conclusion

IVF is an independent risk factor for placental abnormalities and placental-related complications, and the risk is associated with maternal age. It is imperative for the reproductive and obstetric practitioners to carefully consider the impact of IVF treatment on maternal complications and perinatal health outcomes and continue to evaluate the long-term health impact of IVF to ensure the safety and health of the mother and child.

## Data Availability Statement

The original contributions presented in the study are included in the article/**Supplementary Material**. Further inquiries can be directed to the corresponding authors.

## Ethics Statement

The studies involving human participants were reviewed and approved by Peking University Third Hospital Medical Science Research Ethics Committee. Written informed consent for participation was not required for this study in accordance with the national legislation and the institutional requirements.

## Author Contributions

JQ and YW conceived and designed the study. FK, YF, HS and YW did the statistical analyses and drafted the manuscript. RL and YZ contributed to the interpretation of the data. All authors reviewed and revised the manuscripts and supplementary files. All authors read the final manuscript and approved submission.

## Funding

This work was supported by grants from the Beijing Municipal Science & Technology Commission (grant number Z191100006619086 [YW]), and the National Natural Science Foundation of China (grant number 81730038 [JQ]). The funders had no role in the design and conduct of the study; collection, management, analysis, and interpretation of the data; preparation, review, or approval of the manuscript; or decision to submit the manuscript for publication.

## Conflict of Interest

The authors declare that the research was conducted in the absence of any commercial or financial relationships that could be construed as a potential conflict of interest.

## Publisher’s Note

All claims expressed in this article are solely those of the authors and do not necessarily represent those of their affiliated organizations, or those of the publisher, the editors and the reviewers. Any product that may be evaluated in this article, or claim that may be made by its manufacturer, is not guaranteed or endorsed by the publisher.
