# Supplementary material for: Placental Abnormalities and Placenta-Related Complications Following In-Vitro Fertilization: Based on National Hospitalized Data in China
Source: Front Endocrinol (Lausanne). 2022 Jun 30;13:924070. doi: 10.3389/fendo.2022.924070 (PMC9279699; doi:10.3389/fendo.2022.924070)
Supplement: Supplementary file 1 [file DataSheet_1.docx]

**Table S1: Variables and the ICD codes used in this study**

| **Variables** | **ICD codes** |
| --- | --- |
| **Characteristics of pregnancies** | |
| In-vitro fertilization (IVF) | Z31.200, Z31.201, Z37.002, Z37.204, Z37.303, Z37.502, 69.9200x004, 69.9200x006, 69.9202 |
| **Chronic diseases before pregnancy** | |
| Chronic hypertension | O10.000, O10.001, O10.400, O10.401, O10.900 |
| Diabetes | O24.000, O24.100, O24.200, O24.300, O24.301 |
| Thyroid diseases | O99.217, O99.216, O99.215, O99.218, O99.219 |
| Anemia | O99.000, O99.003, O99.004, O99.005, O99.006, O99.007 |
| Circulatory diseases | O10.100, O10.101, O10.300, O10.301, O99.400, O99.419, O99.420, O99.428, O99.429, O99.401, O99.432, O99.430, O99.431, O99.433, O99.409, O90.300, O99.415, O99.408, O99.421, O99.413, O99.416, O99.417, O99.414, O99.402, O75.403, O99.403, O99.418, O99.422, O99.412, O99.410, O99.423, O99.424, O99.406, O99.407, O99.411, O99.404, O99.405 |
| Other diseases (coagulation disorders, kidney diseases, diseases of connective tissues, diseases of respiratory system, and diseases of digestive system) | O99.002, O99.101, O99.102, O99.103, O99.104, O99.105, O99.106, O99.107, O99.108, O99.100, O26.801, O26.802, O90.400, O26.803, O26.804, O26.805, O23.000, O23.001, O23.100, O23.101, O23.200, O23.300, O23.400, O23.900, O23.901, O99.806, O86.200, O86.201, O86.300, O99.811, O99.812, O99.109, O99.805, O99.813, O99.501, O99.502, O99.503, O99.504, O99.505, O99.506, O99.510, O99.511, O99.512, O99.507, O99.509, O99.508, O99.500, O99.600, O99.601, O99.602, O99.603, O99.604, O99.605, O99.606, O99.607, O99.608, O99.609, O99.610, O99.611, O99.612, O99.613, O99.614, O99.615, O99.616, O99.617, O99.618, O99.619, O99.620, O99.621, O99.622, O99.623, O99.624, O26.600, O26.601, O26.602, O26.603, O26.604, O26.605 |
| **Placental abnormalities** | |
| Placenta previa | O44.000, O44.001, O44.002, O44.003, O44.100, O44.101, O44.102, O44.103 |
| Placental abruption | O45.000, O45.001, O45.800, O45.801, O45.900 |
| Placenta accrete | O72.001 |
| Abnormal morphology of placenta | O43.100, O43.101, O43.102, O43.103, O43.104, O43.105, O43.106, O43.107, O43.108, O43.109, O43.110, O43.111, O43.112, O43.800, O43.801, O43.802, O43.803, O43.804, O43.805, O43.800x006, O43.800x007, O43.800x009, O43.900 |
| **Placenta-related complications** | |
| Gestational hypertension | O16.x00, O12.000, O12.100, O12.200, O13.x00, O13.x01 |
| Preeclampsia | O13.x02, O14.000, O14.100, O14.102, O14.900 |
| Eclampsia | O15.000, O15.001, O15.100, O15.101, O15.200, O15.201, O15.900 |
| Gestational diabetes | O24.400, O24.900 |
| Preterm birth | O60.000, O60.001, O60.100, O60.300, P07.300, P59.000, P61.200 |
| Fetal distress | O36.302, O36.305, O36.304, O36.301, O68.003, O68.100, O68.101, O68.200, O68.201, O68.900, O36.300, P20.900 |
| Fetal growth restriction (FGR) | O36.500, O36.504, O36.503, P05.000, P05.100, P05.101, P05.102, P05.200, P05.201, P05.900 |

**Table S2: Detailed categories of abnormal morphology of placenta**

| **Disease** | **ICD Code** | **non-IVF**  **(n=16,352,793)** | **IVF**  **(n=183,059)** | **Total**  **(n=16,535,852)** |
| --- | --- | --- | --- | --- |
| Malformation of placenta | O43.100 | 1789 (0.01) | 34 (0.02) | 1823 (0.01) |
| Velamentous placenta | O43.101 | 53092 (0.32) | 2377 (1.30) | 55469 (0.34) |
| Succenturiate placenta | O43.102 | 32422 (0.20) | 676 (0.37) | 33098 (0.20) |
| Huge placenta | O43.103 | 6021 (0.04) | 123 (0.07) | 6144 (0.04) |
| Circumvallate placenta | O43.104 | 4983 (0.03) | 71 (0.04) | 5054 (0.03) |
| Battledore placenta | O43.105 | 141157 (0.86) | 3654 (2.00) | 144811 (0.88) |
| Placenta triplex | O43.106 | 16403 (0.10) | 294 (0.16) | 16697 (0.10) |
| Bilobed placenta | O43.107 | 5909 (0.04) | 155 (0.08) | 6064 (0.04) |
| Aging placenta | O43.108 | 33059 (0.20) | 514 (0.28) | 33573 (0.20) |
| Placental cyst | O43.109 | 3624 (0.02) | 104 (0.06) | 3728 (0.02) |
| Placenta angioma | O43.110 | 5341 (0.03) | 126 (0.07) | 5467 (0.03) |
| Placental abnormalities | O43.111 | 3882 (0.02) | 109 (0.06) | 3991 (0.02) |
| Placenta circummarginata | O43.112 | 2149 (0.01) | 32 (0.02) | 2181 (0.01) |
| Specific placental complications | O43.800 | 6598 (0.04) | 211 (0.12) | 6809 (0.04) |
| Placental infarction | O43.801 | 5835 (0.04) | 162 (0.09) | 5997 (0.04) |
| Placental necrosis | O43.802 | 671 (0.00) | 12 (0.01) | 683 (0.00) |
| Placental dysfunction | O43.803 | 8326 (0.05) | 192 (0.10) | 8518 (0.05) |
| Placental fibrosis | O43.804 | 10039 (0.06) | 262 (0.14) | 10301 (0.06) |
| Placental hematoma | O43.805 | 4245 (0.03) | 190 (0.10) | 4435 (0.03) |
| Retrochorionic hematoma | O43.800x006 | 7 (0.00) | 1 (0.001) | 8 (0.00) |
| Placental blood sinus | O43.800x007 | 1109 (0.01) | 58 (0.03) | 1167 (0.01) |
| Subamniotic hematoma | O43.800x009 | 11 (0.00) | 0 (0.00) | 11 (0.00) |
| Other placental complications | O43.900 | 6832 (0.04) | 140 (0.08) | 6972 (0.04) |

**Table S3 Sensitivity analysis by performing different regression modelling approaches (Poisson and Logistic regression models)**

|  | **Total**  **(n=16,535,852)** | **IVF**  **(n=183,059)** | **non-IVF**  **(n=16,352,793)** | **aRR (95% CI)** | **aOR (95% CI)** |
| --- | --- | --- | --- | --- | --- |
| **Placental abnormalities** |  |  |  |  |  |
| Placenta previa | 328773 (1.99) | 8865 (4.8) | 319908 (2.0) | **1.87 (1.83 to 1.91)** | **1.93 (1.89 to 1.97)** |
| Placental abruption | 127427 (0.77) | 1853 (1.0) | 125574 (0.8) | **1.16 (1.11 to 1.21)** | **1.16 (1.11 to 1.22)** |
| Placenta accreta | 326875 (1.98) | 8554 (4.7) | 318321 (2.0) | **2.00 (1.96 to 2.04)** | **1.64 (1.62 to 1.66)** |
| Abnormal morphology of placenta | 308626 (1.87) | 8231 (4.5) | 300395 (1.8) | **2.12 (2.07 to 2.16)** | **2.17 (2.13 to 2.22)** |
| **Placenta-related complications** |  |  |  |  |  |
| Gestational hypertension | 276136 (1.67) | 5962 (3.3) | 270174 (1.7) | **1.55 (1.51 to 1.59)** | **1.57 (1.53 to 1.61)** |
| Preeclampsia | 456977 (2.76) | 10082 (5.5) | 446895 (2.7) | **1.54 (1.51 to 1.57)** | **1.59 (1.56 to 1.62)** |
| Eclampsia | 20621 (0.12) | 226 (0.1) | 20395 (0.1) | 0.91 (0.80 to 1.04) | 0.91 (0.80 to 1.04) |
| Preterm | 808063 (4.89) | 15066 (8.2) | 792997 (4.9) | **1.48 (1.46 to 1.51)** | **1.53 (1.51 to 1.56)** |
| Fetal distress | 1010414 (6.11) | 14627 (8.0) | 995787 (6.1) | **1.39 (1.37 to 1.42)** | **1.43 (1.41 to 1.45)** |
| FGR | 134722 (0.81) | 2205 (1.2) | 132517 (0.8) | **1.36 (1.30 to 1.42)** | **1.37 (1.31 to 1.43)** |

IVF: in vitro fertilization; Non-IVF: non-in vitro fertilization; FGR: fetal growth restriction.

RR: relative risk; aRR: adjusted relative risk. OR: odd ratio; aOR: adjusted odd ratio; CI: confidence interval.

aRR were calculated in Poisson regression modelling with restricted cubic splines of exact maternal age, with adjustment for geographic region, maternal age, year, ethnicity, and chronic diseases before pregnancy.

aOR were calculated in Logistic regression modelling with restricted cubic splines of exact maternal age, with adjustment for geographic region, maternal age, year, ethnicity, and chronic diseases before pregnancy.
